# Dietary intake adequacy among Iranian older adults: evidence from national household survey data and two population-based cohorts

**DOI:** 10.3389/fnut.2026.1808784

**Published:** 2026-06-26

**Authors:** Ali Shokri, Nasrin Omidvar, Delaram Ghodsi, Hassan Eini-Zinab, Farshad Sharifi, Arezoo Rezazadeh

**Affiliations:** 1 Department of Community Nutrition, National Nutrition and Food Technology Research Institute, Faculty of Nutrition Sciences and Food Technology, Shahid Beheshti University of Medical Sciences, Tehran, Iran; 2 Department of Nutrition Research, National Nutrition and Food Technology Research Institute and Faculty of Nutrition Sciences and Food Technology, Shahid Beheshti University of Medical Sciences, Tehran, Iran; 3 Elderly Health Research Center, Endocrinology and Metabolism Population Sciences Institute, Tehran University of Medical Sciences, Tehran, Iran

**Keywords:** older adults, Iran, macronutrient, micronutrient, mean adequacy ratio, mean energy intake, nutrient adequacy ratio

## Abstract

**Introduction:**

Iran’s aging population faces significant nutritional challenges. Comprehensive data on the dietary adequacy of older adults (OAs) using multiple sources are limited. This study assessed the adequacy of energy, macro- and micronutrient intake among Iranian OAs using data from the national household survey and two population-based cohorts.

**Methods:**

To comprehensively assess dietary intake patterns and nutrient adequacy among Iranian OAs, a multi-source analysis was conducted using: (1) the Iranian Household Expenditure and Income Survey (IHEIS, 2019–2022, *n* = 110,765 OAs) to estimate population-level intake via the Older Adult Male Equivalent (OAME) method; and (2) individual-level data from two cohorts, the Tehran Lipid and Glucose Study (TLGS, *n* = 1,839) and the Birjand Longitudinal Aging Study (BLAS, *n* = 1,325). Nutrient Adequacy Ratios (NAR) and Mean Adequacy Ratios (MAR) were calculated. Due to methodological differences, the datasets were analyzed separately, and the findings were interpreted in a complementary manner.

**Results:**

IHEIS estimates revealed low intakes of fruits, vegetables, dairy, and meat, alongside a grain-dominant dietary pattern. Mean energy intake was below recommended levels and declined with age. Severe and persistent inadequacy was observed in the intake of several micronutrients, particularly vitamin D (NAR ≤ 0.04), vitamin A (NAR < 0.25), calcium (NAR 0.22–0.35), zinc (NAR 0.25–0.39), folate, and vitamin B12. MAR values ranged from 0.51 to 0.61, indicating that only about half of cumulative nutrient requirements were met, with consistently lower adequacy among women. Cohort data showed higher mean energy and nutrient intakes than those in IHEIS; however, substantial inter-individual variability was evident, and inadequacies in calcium, vitamin D, folate, and vitamin B12 persisted across cohorts.

**Discussion:**

The diet of Iranian OAs is characterized by low dietary diversity, a high reliance on grains, and critical inadequacies in multiple micronutrients, with notable disparities by sex and age. Integrating adequacy-based indicators with multiple data sources provides a more accurate assessment of dietary vulnerability in older populations. The convergence of findings from household and individual-level data underscores an urgent need for targeted nutritional interventions to support healthy aging in Iran.

## 1 Introduction

Population aging is set to be one of the greatest transformations of the twenty-first century, with far-reaching implications for all sectors of society. It is estimated that by 2050, one in five people will be aged 60 or older (1). While most will be in good health, this population will be confronted with the potential effects of the aging process, which can result in decreased quality of life, illness, or chronic diseases (2). Research over the past few decades has consistently demonstrated that adequate nutritional status can play a key role in preventing or delaying the progression of age-related diseases, including cardiovascular diseases (CVDs), reduced cognitive function, and osteoporosis (3). Nutrition not only supports physical health but also influences psychological well-being, functional independence, and longevity, making it a cornerstone of healthy aging strategies (4).

However, physiological and social changes, such as decreased food intake, impaired sensory perception, malabsorption, reduced activity, and increased disability, can heighten the risk of inadequate nutritional status in this population (5). These challenges are often accompanied by socioeconomic inequalities, limited access to nutrient-dense foods, and cultural dietary patterns, all of which increase vulnerability to malnutrition (6). As a result of these changes, the recommended values for some nutrients are modified in the older adults (OAs), with higher requirements for protein, calcium, vitamin D, B12, and certain antioxidants to compensate for age-related metabolic decline and bone loss (7).

The elderly population faces significant nutritional challenges, including both undernutrition and overnutrition. A study in Iran found that the prevalence of malnutrition among the elderly was almost 12% across socioeconomic groups (8). Since low energy intake is often difficult to diagnose, elderly individuals are at a higher risk of weight loss, nutritional deterioration, morbidity, and mortality. Conversely, obesity also presents a major health risk; a systematic review highlighted an obesity prevalence of 21.4% among adults aged over 60 years, underscoring their vulnerability to chronic diseases such as cardiovascular disease (9). Beyond excessive caloric intake, micronutrient deficiencies pose another critical challenge to the health and well-being of the aged population.

Given the global trend of population aging and the broad implications of inadequate dietary intake among this group in Iran, there is a pressing need for comprehensive, well-designed studies to assess dietary adequacy relative to the requirements recommended for OAs. Accurate evaluation of nutrient intake, comparison with dietary reference intakes, and identification of influencing factors can provide valuable insights for designing preventive programs and informing policy decisions at national and regional levels.

Despite growing concern about nutritional inadequacy among Iranian OAs, existing evidence remains fragmented and methodologically limited. Most studies have focused either on the general adult population or on average dietary intakes, without assessing adequacy relative to age-specific requirements. Moreover, the potential discrepancies between household-based dietary estimates and individual-level intake data have not been systematically examined in this age group in Iran. Accordingly, the present study aims to evaluate the nutritional adequacy of dietary intake among OAs using data from the national household expenditure and income survey and two population-based cohorts. Addressing these gaps is essential to generating evidence that can effectively inform nutrition policy and aging-related interventions.

## 2 Materials and methods

### 2.1 Study design and data sources overview

This study is a secondary, descriptive-comparative analysis of nationally representative household data and two population-based cohort studies. The present study employed a multi-source comparative approach to describe dietary intake and nutrient adequacy among Iranian OAs. Given the absence of a single national dataset that provides both detailed individual dietary intake and nationally representative consumption data, two complementary sources were analyzed in parallel.

First, data from the Iranian National Household Expenditure and Income Survey (IHEIS), a nationally representative household-based survey, were used as a proxy of usual intake at the population level to estimate average dietary and nutrient intakes of OAs. Household-level food acquisition data were converted to individual-equivalent intakes using the older adult male equivalent (OAME) approach, enabling estimation of population-level mean intakes rather than precise individual consumption estimates.

Second, individual-level dietary intake and nutrient adequacy were assessed using two population-based cohort studies, the Tehran Lipid and Glucose Study (TLGS) and the Birjand Longitudinal Aging Study (BLAS), which provide detailed and direct measurements of individual food and nutrient intake. Due to fundamental differences in data structure and the level of dietary assessment (household-based versus individual-based), the datasets were analyzed separately and not pooled. Findings from each source are presented side by side and interpreted in a complementary manner to provide a coherent picture of average intake patterns and individual-level nutrient adequacy among OAs in Iran.

### 2.2 Analysis 1: Estimation of dietary intake from household expenditure data (IHEIS)

The IHEIS is an annual survey carried out by the Statistical Center of Iran (SCI). It employs a stratified, three-stage, clustered sampling design to select participants residing in both urban and rural areas nationwide. To obtain more accurate estimates, the samples were equally distributed across all months of the year between 2019 and 2022 and the total sample size of OAs (>60 years) reached 110,765. This study used data from IHEIS, which provides an integrated overview of household structure and economic behavior. The dataset encompasses four principal domains; (1) demographic and social characteristics of household members, including the number of members and detailed demographic information such as age, sex, educational attainment, employment status, and marital status; (2) housing and living conditions, covering characteristics of the residence and availability of domestic facilities; (3) patterns of consumption and expenditure, encompassing both food and non-food expenditures as indicators of resource allocation across different sectors; (4) income composition, detailed information on earnings from wages, self-employment, and other income sources.

#### 2.2.1 Food data

Since the food consumption data were collected at the household level, they were converted into individual-level dietary intakes. To avoid bias in the per capita estimates, the OAME was calculated to account for differences in energy requirements among household members (10). OAME is defined as the ratio of each household member’s estimated energy requirement to that of a reference male aged 60 years or older, both with moderate physical activity. Both the reference value and household members’ energy requirements were derived in accordance with recommendations from the Food and Agriculture Organization (FAO) and the World Health Organization (WHO) (11, 12).

Unlike crude per capita estimates, this approach allows the identification of each household member’s contribution to household food expenditure. Using this method, the total OAME for each household was calculated by summing the OAME values of all household members, accounting for their age and sex. Therefore, each person’s share of the household’s total OAME was calculated by dividing each person’s OAME by the household’s total OAME and multiplying by the quantity of each food item, after separating people aged 60 and older.

Given that a portion of purchased foods is typically wasted, the actual consumption quantities were adjusted using FAO estimates of food loss percentages for each food group at the consumption stage within the “supply-to-consumption” chain (13). Following this adjustment, cooking yield factors were applied to food items that underwent processing or heat exposure, using Iran-specific values (14). The energy, macronutrient, and micronutrient contents of foods were subsequently calculated using NUTRITIONIST IV software, which was updated for Iranian foods.

#### 2.2.2 Dietary adequacy

To assess the adequacy of nutrient intake among men and women, the daily intakes of individual nutrients were compared with age-and sex-specific recommendations from WHO/FAO (2004). Then, the Nutrient Adequacy Ratio (NAR) index was defined according to the guidelines of the INNDEX protocol (2018), by dividing the actual intake of each nutrient by its corresponding recommended intake:


$$\mathrm{NAR}=\frac{\text{Individual nutrient intake}}{\mathrm{Age}\;\text{and}\;\mathrm{sex}-\text{specific}\;\mathrm{RDA}\;}$$

Mean adequacy index (MAR) was then computed as an overall indicator of dietary adequacy, using NAR values of 18 macro- and micronutrients, including energy, protein, carbohydrate, linoleic acid, linolenic acid, vitamin A, vitamin C, thiamine, riboflavin, niacin, pyridoxine, folic acid, cobalamin, vitamin D, iron, zinc, calcium, and phosphorus. Each NAR value was truncated at 1 to prevent compensation for inadequacy in one nutrient by excessive intake of another. Subsequently, MAR was derived by averaging all NAR values.


$$\mathrm{MAR}=\frac{\sum \mathrm{NAR}\;(\text{trancuated to}\;1)}{\text{number of nutrients}}$$

### 2.3 Analysis 2: Nutrient intake from cohort studies (TLGS and BLAS)

#### 2.3.1 Cohorts descriptions and participants

This study also utilized secondary data from two independent population-based cohort studies conducted in Iran: Tehran Lipid and Glucose Study (TLGS) and Birjand Longitudinal Aging Study (BLAS). Both datasets were used to assess individual-level dietary intake and nutrient adequacy among OAs aged 60 years and older. From the TLGS dataset, individual-level dietary information of 1,839 OAs residing in Tehran was extracted and included in the analysis. For the BLAS cohort, data representing 1,325 urban OAs in Birjand city, South Khorasan, eastern Iran, from the first wave of the cohort (October 2018 and April 2019)—including means and standard deviations of key variables—were provided by the respective research team. Participants in the BLAS cohort were recruited using a multistage, stratified, cluster-random sampling method targeting individuals aged 60 years and over.

#### 2.3.2 Dietary assessment and data processing

In both cohorts, habitual dietary intake was recorded using a validated Food Frequency Questionnaire (FFQ) developed specifically for the Iranian population (15). This FFQ included major food items consumed in Iran and allowed estimation of daily energy, macronutrient, and micronutrient intakes. Dietary data were reported in both cohorts as mean daily intakes (g/day). For data preparation, individual-level data from the TLGS cohort were first screened for completeness. Records with more than 20% missing information on food items or nutrient values were excluded. Outliers were identified and removed based on the acceptable energy intake range (500–5,000 kcal per day) and values exceeding three standard deviations from the mean for major nutrients. For the BLAS cohort, since the data were provided only as summary statistics (means and standard deviations), individual-level data cleaning was not possible, and analyses were performed directly on the provided summary data. The BLAS summary data included the same set of parameters and were incorporated directly into the results tables. Due to methodological heterogeneity across studies, such as assessment tools and measurement levels (household or individual), datasets were not pooled. Therefore, the analysis was performed independently.

### 2.4 Statistical analysis

For the IHEIS, all demographic calculations (OAME factors, total household OAME, individual shares) were performed using Microsoft Excel and R software. The primary output was a dataset in which each OA (≥60 years) had an estimated daily quantity (in grams) of each food item, adjusted for waste and cooking yield, as described in Sections 2.2.2 and 2.2.3. Monthly purchase quantities were converted to daily values by dividing by 30.5, reflecting the average length of a month in the Iranian calendar (in which six months have 30 days and six have 31). Descriptive statistics (mean, standard deviation, and 95% confidence interval) for daily per-older-adult food quantities were calculated overall and stratified by age groups (60–74, 75–84, and ≥85 years) and sex. For the TLGS cohort, all analyses (descriptive statistics and mean nutrient intakes) were performed on the cleaned individual-level data using SPSS (version 26). For the BLAS cohort, analyses were based on the provided data. Direct comparisons of means to RDAs were made using the provided mean intakes. Descriptive statistics (mean, standard deviation, and 95% confidence interval) for the daily per-older-adult food quantities were calculated overall and stratified by sex.

### 2.5 Ethical considerations

Both TLGS and BLAS protocols had previously been approved by the ethics committees of their respective institutions. All participants had completed written informed consent prior to enrollment. The IHEIS data used in the present study were secondary and contained no identifying information.

## 3 Results

### 3.1 Analysis 1: Estimated daily dietary intake from household purchases (IHEIS)

#### 3.1.1 Food groups intake patterns

Based on the IHEIS data (Table 1), the estimated daily OA consumption for several key food groups was considerably low, with substantial variability indicated by large standard deviations. The mean (±SD) estimated consumption of fruits and vegetables was 58.1 ± 70.8 g/day and 93.1 ± 96.0 g/day, respectively; both below the recommendations. The standard deviation for fruits exceeding the mean suggests extreme variability across participants from households. The estimated dairy product intake was markedly low (64.1 ± 68.0 g/day). The near-equal magnitude of the mean and standard deviation underscores a highly unequal distribution. Similarly, the estimated intake of meat products averaged 55.6 ± 63.5 g/day, which does not meet the recommended dietary intake for OAs. In contrast, the estimated grain consumption was high on average (347.6 ± 292.9 g/day) and exhibited substantial absolute variability. High grain intake indicates the dominant role of bread and the grain group in the dietary pattern of elderly Iranians.

**Table 1:** Mean daily intake of major food groups among Iranian community-dwelling older adults compared with national household survey data, Tehran Lipid and Glucose Study (TLGS), Birjand Longitudinal Aging Study (BLAS), and dietary recommendations.

Food groups	Individual-level dietary intake				Household-level dietary intake		Nutritional recommended values
	BLAS (*n* = 1,325)		TLGS (*n* = 1,839)		IHEIS (2019–2022) (*n* = 110,766)		
	Mean	SD	Mean	SD	Mean	SD	
Fruits (g)	56.2	104.5	384.5	299.0	58.1	70.8	2 cups eq/day (210 g/day) (30)
Vegetables (g)	108.5	70.2	299.8	171.8	93.1	96.0	2.5 cups eq/day (190 g/day) (30)
Grains (g)	-	-	377.6	175.7	347.6	292.9	6 ounces eq/day (168 g/day) (30)
Meat and egg (g)	37.4	42.0	69.1	49.0	55.6	63.5	3.5 ounces eq/day (98 g/day) (30)
Dairy (g)	115.3	219.0	278.7	184.6	64.1	68.0	3 cups eq/day (513 g/day) (30)

Data sources include the Birjand Longitudinal Aging Study (BLAS), Tehran Lipid and Glucose Study (TLGS), and the Iran national Household Expenditure and Income Survey (IHEIS; 2019–2022).

Recommended intakes are expressed as cup equivalents (c-eq/day) or ounce equivalents (oz-eq/day) according to established dietary guidelines for older adults and converted to grams for comparison where applicable.

Grain intake data were not available for BLAS.

The findings from individual and household-levels should be interpreted complementarily, due to their distinct methodological approaches.

#### 3.1.2 Energy, macro- and micronutrients intake

Based on the IHEIS data (Table 2), the mean (±SD) of estimated daily energy intake for OAs was 1447.6 ± 1066.0 kcal, which is below the recommended levels for both men and women. This estimate showed substantial variability, as indicated by the large standard deviation. The contributions of proteins and fats to total energy were at the lower end of recommended ranges: carbohydrates provided 65%, proteins 12.6%, and total fats 24%. Notably, dietary fiber intake was critically low, with a mean (±SD) of 9.2 ± 7.2 g/day, far below the sex-specific recommendations of 21–30 g/day.

**Table 2:** Mean daily intake of energy, macro- and micronutrients among Iranian community-dwelling older adults compared with national household survey data, Tehran Lipid and Glucose Study (TLGS), Birjand Longitudinal Aging Study (BLAS), and dietary reference values.

Energy & macronutrients	Individual-level dietary intake				Household-level dietary intake		Nutritional recommended values
	BLAS (*n* = 1,325)		TLGS (*n* = 1,839)		2019–2022 (*n* = 110,766)		
	Mean	SD	Mean	SD	Mean	SD	
Energy (Kcal)	2466.4	1535.1	1938.2	633.9	1447.6	1066.0	Men: 2,450 kcal/day‡ Women: 2,000 kcal/day‡ (68)
Carbohydrate (g) (En%)	356.7 (58)	309.0	300.1 (62)	106.0	234.2 (65)	170.4	55–75 En% (69)
Protein (g) (En%)	83.0 (13.4)	50.0	73.3 (15)	37.7	49.3 (12.6)	39.2	10–35 En% (70)
Fat (g) (En%)	85.7 (31)	38.4	60.5 (28)	26.2	46.1 (24)	45.0	20–35 En% (71)
SFAs (g) (En%)	30.5 (11)	14.51	19.1 (9)	9.0	11.5 (5.5)	15.9	10 En% (71)
MUFAs (g) (En%)	29.0 (10)	13.8	20.9 (10)	10.3	17.4 (8.7)	28.4	15–20 En% (71)
PUFAs (g) (En%)	16.0 (6)	9.4	12.6 (6)	5.8	12.7 (7.8)	14.0	6–11 En% (71)
Fiber intake (g)	13.89	13.6	37.1	18.0	9.2	7.2	Men: 30 g/day Women: 21 g/day (72)
Minerals							
Calcium (mg)	1024.9	621.8	923.3	404.3	353.0	317.0	1,200 mg/day (73)
Iron (mg)	20.5	17.2	19.4	15.2	10.6	8.4	8 mg/day (72)
Zinc (mg)	12.7	9.0	11.2	7.5	3.6	3.7	Men: 11 mg/day Women: 8 mg/day (72)
Phosphorous (mg)	1647.5	1126.5	1314.4	470.2	406.0	391.4	700 mg/day (72)
Vitamins							
Vitamin A (μg)	-	-	680.5	1257.5	271.4	375.0	Men: 900 μg/day Women: 700 μg/day (72)
Vitamin B1 (mg)	-	-	1.7	1.1	1.3	1.14	Men:1.2 mg/day Women: 1.1 mg/day (72)
Vitamin B2 (mg)	-	-	0.6	0.8	0.8	1.2	Men: 1.3 mg/day Women: 1.1 mg/day (72)
Vitamin B3 (mg)	-	-	19.6	7.6	17.1	15.2	Men: 16 mg/day Women: 14 mg/day (72)
Vitamin B6 (mg)	2.0	1.0	1.8	1.8	0.8	0.9	Men: 1.7 mg/day Women: 1.5 mg/day (72)
Vitamin B9 (μg)	-	-	468	159.0	171.3	179.0	400 μg/day (72)
Vitamin B12 (μg)	3.0	1.7	2.7	1.6	1.06	2.3	2.4 μg/day (72)
Vitamin D (μg)	-	-	3	7.5	0.4	0.5	20 μg/day (73)
Vitamin C (mg)	98.7	113.0	139.1	92.7	31.3	26.8	Men: 90 mg/day Women: 75 mg/day (72)

Values are presented as mean ± standard deviation (SD).

Energy contributions of macronutrients are expressed as percentage of total energy intake (En%) where applicable.

Dietary reference values are based on established recommendations for older adults and are presented separately for men and women where applicable.

Missing values indicate nutrients not available in the corresponding dataset.

The estimated intakes of most micronutrients from household food purchases were substantially below recommended levels, with evidence of high variability (large SDs relative to means). Mean (±SD) for calcium intake was 353.0 ± 317.0 mg/day, less than one-third of the recommendation (1,200 mg/day). Zinc intake was similarly inadequate (3.6 ± 3.7 mg/day), whereas iron intake averaged 10.6 ± 8.4 mg/day, exceeding the recommended 8 mg/day. A severe deficiency was observed in fat-soluble vitamins. Vitamin D intake was negligible (0.4 ± 0.5 μg/day), and vitamin A intake was 271.4 ± 375.0 μg/day, both substantially below recommended levels. Among water-soluble vitamins, folate (B9) intake was low (171.3 ± 179.0 μg/day), and vitamin B12 intake was estimated at 1.06 ± 2.3 μg/day. Vitamin C intake was 31.3 ± 26.8 mg/day, which also fell below recommended intakes and showed marked variability across households.

#### 3.1.3 Age-related variation in energy and nutrient intake among OAs

**Figure 1:**
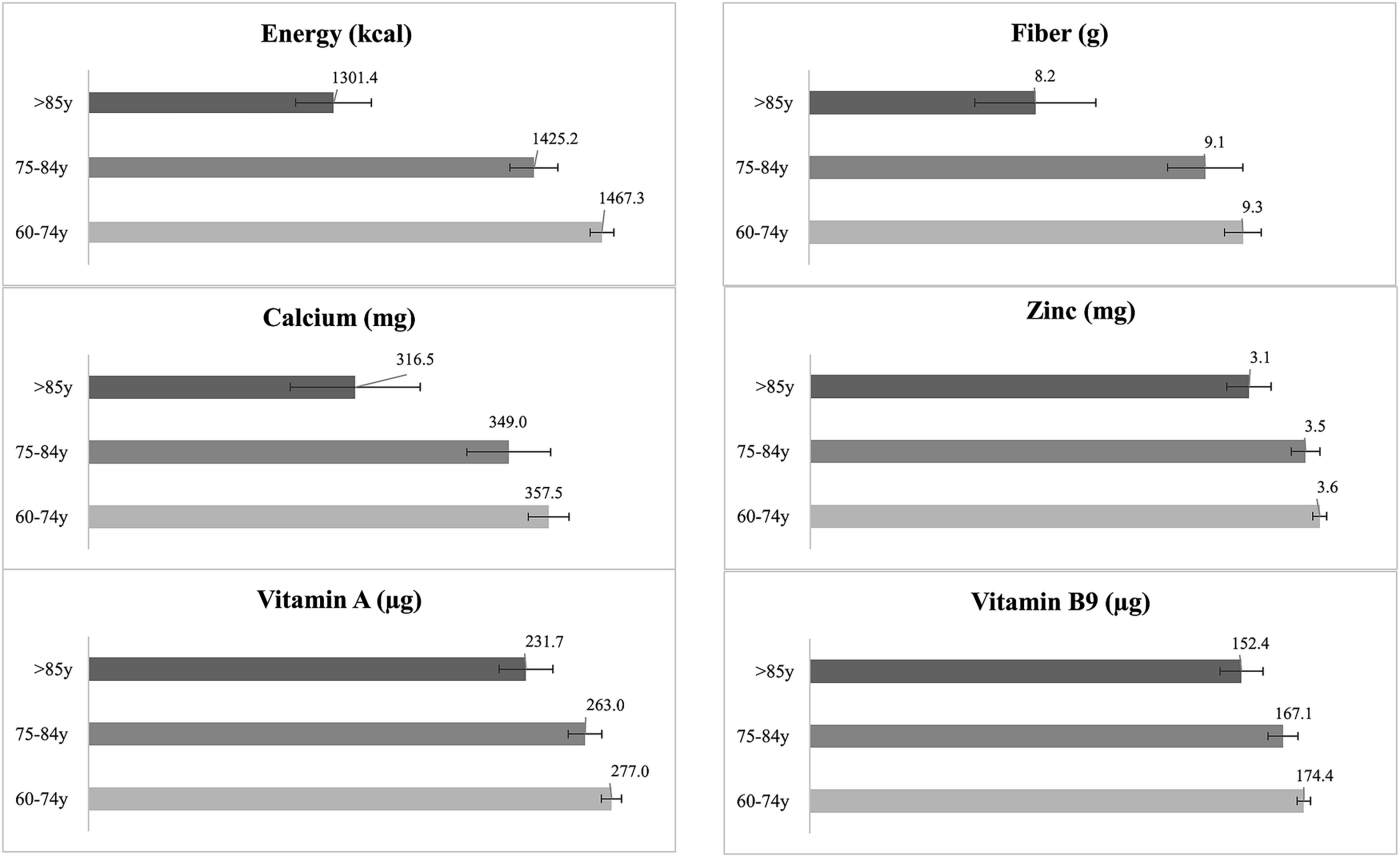
Mean intakes of macro-and micronutrients of Iranian elderly based on IHEIS in older adults age groups from 2019–2022. This figure shows the mean daily intake of energy, macronutrients, fiber, and selected micronutrients in three age groups: 60–74 years (*n* = 81,745), 75–84 years (*n* = 21,728), and ≥85 years (*n* = 7,292). The bars represent the mean daily intake and the error bars represent the 95% confidence interval (95% CI). Data are extracted from the aggregate analysis of the Household Expenditure and Income Survey (IHEIS). Complete results are reported in Supplementary Table 1.

As illustrated in Figure 1, analysis of the IHEIS data demonstrated a progressive decline in mean energy intake and in most nutrients with advancing age among OAs. Mean daily energy intake decreased from 1,467 kcal (95% CI: 1459.8, 1474.2) in the 60–74 age group to 1,425 kcal (95% CI: 1410.1, 1439.9) in the 75–84 age group and further to 1,301 kcal (95% CI: 1277.7, 1324.3) in those aged over 85 years. A similar age-related downward trend was observed for dietary fiber intake. Mean fiber intake declined from 9.3 g (95% CI: 9.20, 9.40) in the youngest group to 9.1 g (95% CI: 8.90, 9.30) in those aged 75–84 years and to 8.2 g (95% CI: 7.88, 8.52) in the oldest group, which is far from the recommended values.

Calcium intake also showed a consistent decrease with age, declining from 357.5 mg (95% CI: 353.2, 361.8) to 349 mg (95% CI: 340.1, 357.9) and 316.5 mg (95% CI: 302.7, 330.3). These values are less than one-third of the recommended daily intake in all age groups. Zinc intake also decreased with age, from 3.6 mg (95% CI: 3.55, 3.65) to 3.5 mg (95% CI: 3.40, 3.60) and 3.1 mg (95% CI: 2.94, 3.26). Vitamin A intake was low across all three age groups and declined steadily. The mean intake of this vitamin declined from 277 μg (95% CI: 271.7, 282.3) in the youngest group to 263 μg (95% CI: 254.1, 271.9) and 231.7 μg (95% CI: 217.6, 245.8) in the elderly aged 85 years and older. Folate (vitamin B9) intake also diminished with age, from 174.4 μg (95% CI: 172.0, 176.8) in the 60–74 age group to 167.1 μg (95% CI: 161.8, 172.4) and 152.4 μg (95% CI: 144.8, 160.0) in people over 85 years. Overall, the simultaneous decrease in intake of energy, fiber, calcium, zinc, and key vitamins with advancing age highlights an increased risk of nutritional inadequacies in OAs. Detailed estimates of nutrient intakes across age groups are presented in Supplementary Table 1.

#### 3.1.4 Sex-related differences in energy and nutrient intake among OAs

**Figure 2:**
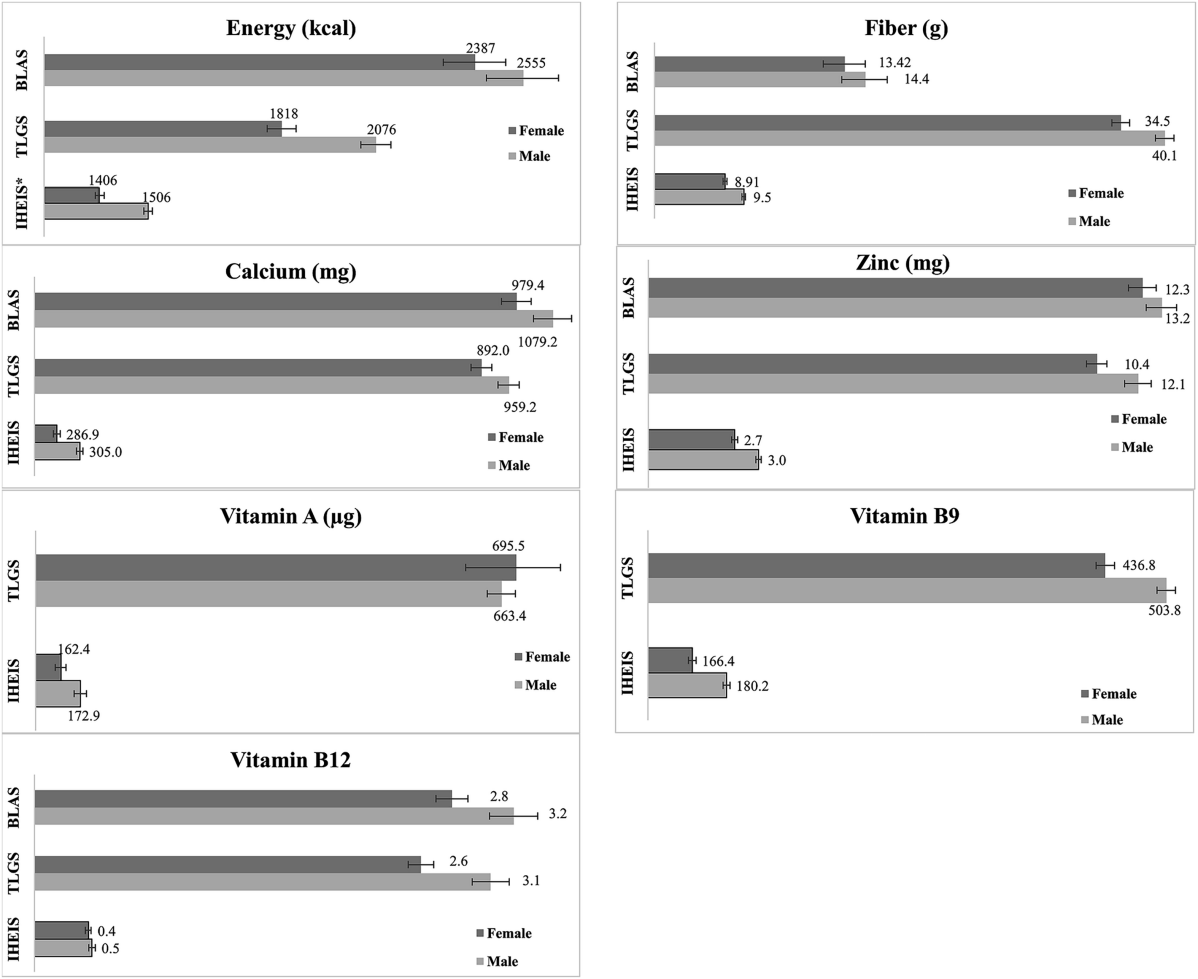
Mean intakes of selected macro and micronutrients of Iranian male and female elderly based on IHEIS, TLGS, and BLAS. This figure shows the mean daily intake of energy and selected macro- and micronutrients among elderly Iranian men and women, based on IHEIS, TLGS, and BLAS data. The height of the bars indicates the mean intake value, and the error bars indicate the 95% confidence interval (95% CI). Sample sizes and complete results are reported in the Supplementary Table 2. *Because of the different methodological approaches, IHEIS (household-level) findings are outlined with a solid black border.

As shown in Figure 2, the IHEIS estimates revealed clear and significant sex-related differences in energy and nutrient intake among OAs. Men had a higher mean daily energy intake (1,506 kcal, 95% CI: 1497.2, 1514.8) than women (1,406 kcal, 95% CI: 1397.0, 1415.0).

This sex-based pattern was consistent across several key nutrients. Mean calcium intake was higher in men (305 mg/day; 95% CI: 302.4–307.6) compared to women (287 mg/day; 95% CI: 284.3–289.7), although intakes in both sexes remained well below recommended levels. Similarly, estimated zinc intake was 3.00 mg (95% CI: 2.97–3.03) per day for men and 2.75 mg (95% CI: 2.72–2.78) per day for women. Sex–related detailed nutrient intakes are provided in Supplementary Table 2.

For vitamin A, the estimated intake was below recommended levels for both sexes, with means of 173 μg (95% CI: 169.6, 176.4) for men and 162 μg (95% CI: 159.2, 164.8) for women. Folate intake followed a similar pattern, with men consuming a mean of 180 μg/day (95% CI: 178.51, 181.49) and women 166 μg/day (95% CI: 164.51, 167.49).

Overall, these findings demonstrate persistent sex-related differences in energy and nutrient intake among Iranian OAs, with men consistently reporting higher intakes than women. However, the inadequacy of several micronutrients remained evident in both sexes, as indicated by household food purchase estimates.

#### 3.1.5 Nutrient adequacy

**Figure 3:**
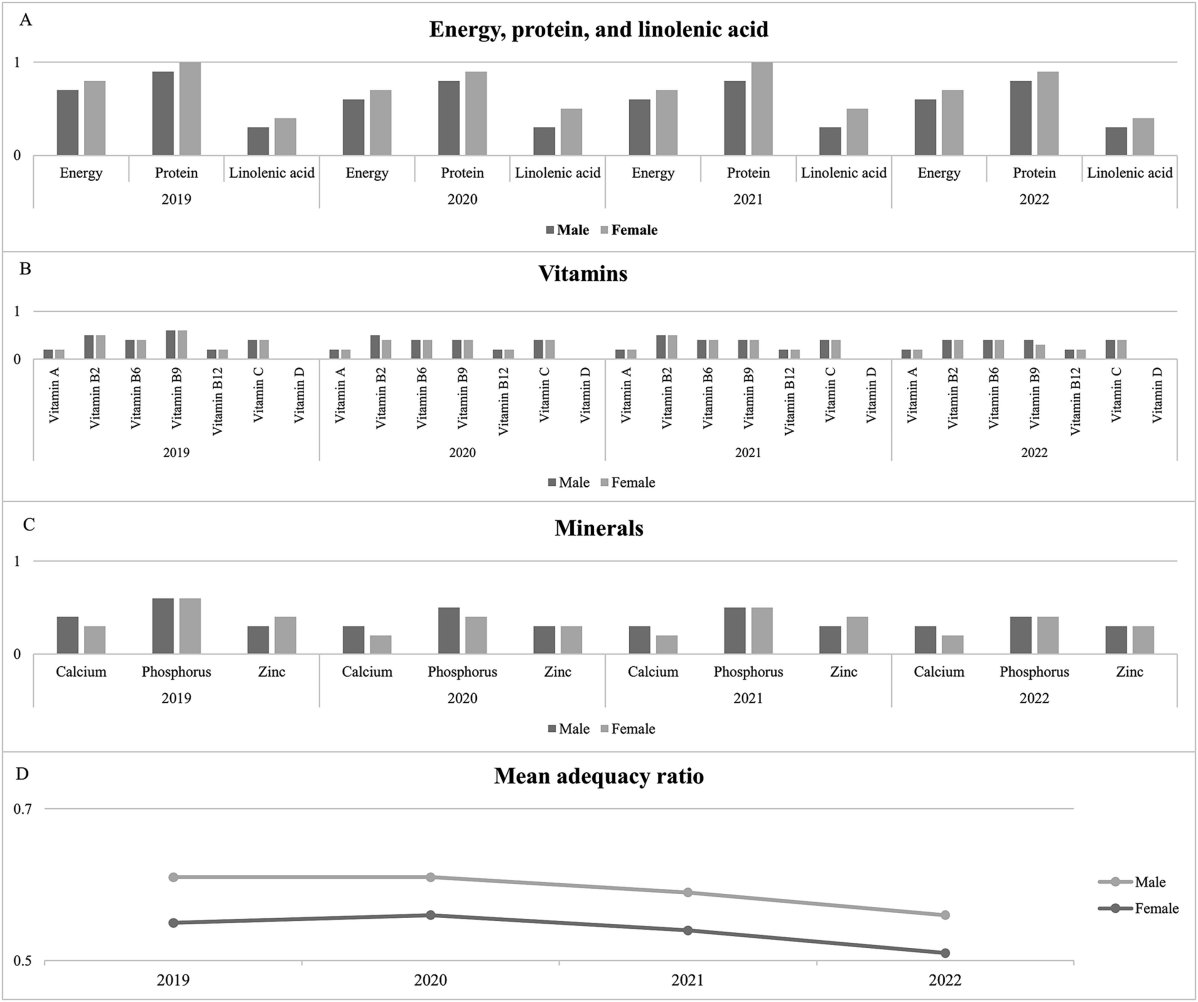
Nutrient Adequacy Ratio (NAR) and Mean Adequacy Ratio (MAR) among Iranian older adults from 2019 to 2022, stratified by sex. **(A)** Selected macronutrients and essential fatty acids (energy, protein, and linolenic acid) for males and females. **(B)** Vitamins (vitamin A, vitamin D, vitamin B2, vitamin B6, vitamin B9, vitamin B12, and vitamin C) for males and females. **(C)** Selected minerals (calcium, phosphorus, and zinc) for males and females. **(D)** Mean Adequacy Ratio (MAR) by year. NAR was calculated as the ratio of nutrient intake to the corresponding recommended intake and truncated at 1.0. MAR was calculated as the mean of NAR values across all assessed nutrients. Protein was additionally included because of its recognized importance for maintaining muscle mass, functional capacity, and healthy aging among older adults. Complete NAR and MAR results for all assessed nutrients are provided in Supplementary Table 3.

As shown in Figure 3, the NAR results revealed a widespread and persistent inadequacy of most micronutrients in the elderly between 2019 and 2022, with a relatively stable pattern over time and between sexes. A severe and uniform inadequacy was observed for fat-soluble vitamins. Vitamin A intake was consistently inadequate in both sexes and in all years, with NAR values of 0.19 in men and between 0.23 and 0.24 in women, indicating that less than a quarter of the recommended intake was achieved. Vitamin D demonstrated the lowest adequacy among the micronutrients. In men, the NAR declined from 0.03 in 2019 to 0.02 in 2020–2022, and in women it remained between 0.02 and 0.04, reflecting severe and persistent inadequacy of this vitamin throughout the study period.

A concerning decline in the adequacy of key minerals was noted. Calcium adequacy was low and declined over time, with the NAR decreasing from 0.35 in 2019 to 0.28 in 2022 in men and from 0.27 to 0.22 in women. Zinc intake was inadequate in both sexes, with NAR values ranging from 0.25 to 0.31 in men and from 0.32 to 0.39 in women, and consistently higher in women than in men.

Among B vitamins, vitamins B1 and B3 indicated relatively adequate intake. However, folate intake declined over time, with NAR values decreasing from 0.64 in 2019 to 0.36 in 2022 in men and from 0.59 to 0.31 in women. In both sexes, the NAR of vitamin B12 also remained below 0.25 in all years (men: 0.18–0.23; women: 0.17–0.22). Overall, the NAR findings indicated inadequate intake of vitamin A, vitamin D, calcium, zinc, folate, and vitamin B12 in the elderly, while iron and most B vitamins were within acceptable intake levels. The MAR results further showed that the elderly’s overall dietary adequacy was below the desired value in all years of the study (Figure 3). In 2019, the MAR values were 0.61 in men and 0.55 in women, with similar values observed in 2020 (0.61 and 0.56, respectively). A slight decrease in overall dietary adequacy was observed, with MAR reaching 0.59 in men and 0.54 in women in 2021. This downward trend intensified in 2022, with the MAR for men decreasing to 0.56 and for women to 0.51. In all years of the study, the MAR value in women was consistently lower than that of men. Overall, MAR values ranged from 0.51 to 0.61, indicating that, on average, only about 51–61% of the cumulative adequacy of the studied micronutrients was provided in the elderly diet. Complete NAR and MAR results for all assessed nutrients are presented in Supplementary Table 3.

### 3.2 Analysis 2: Estimated daily dietary intake from cohort studies (TLGS and BLAS)

#### 3.2.1 Food group intake patterns

Dietary intake patterns from the two cohort studies are presented in Tables 1, 2, revealing considerable within-cohort variability.

In the TLGS cohort, mean (±SD) daily intake of fruits and vegetables was 384.5 ± 299.0 g/day and 299.8 ± 171.8 g/day, respectively. The very large standard deviation for fruit intake highlights wide individual variation. In the BLAS cohort, the intake was 56.2 ± 104.5 g/day for fruits and 108.5 ± 70.2 g/day for vegetables. The standard deviation for fruit intake in BLAS is nearly double the mean, indicating a highly skewed distribution. Dairy consumption showed high variability in both cohorts: 278.7 ± 184.6 g/day in TLGS and 115.3 ± 219.0 g/day in BLAS.

#### 3.2.2 Energy, macro- and micronutrient intake

As shown in Table 2, mean daily energy and nutrient intakes differed between the TLGS and BLAS cohorts, with notable variability in both cohorts. In the BLAS cohort, reported energy intake was higher (2,466 kcal/day) than in TLGS (1938 kcal/day). Despite differences in daily energy intake, the contributions of macronutrients to total energy intake (carbohydrates, protein, and fat) fell within recommended ranges in both cohorts. Dietary fiber intake in TLGS (37 g/day) was significantly higher than in BLAS (14 g/day).

Regarding mineral intake, calcium intake was below the recommended level (1,200 mg/day) in both cohorts. In contrast, iron intake exceeded the recommended amount in both studies. In the TLGS cohort, vitamin D intake was critically low. Vitamin A intake in this study exhibited substantial variability. Intake of B vitamins showed mixed patterns. The key finding of both studies was the high variability in nutrient intake, indicating that mean values conceal a wide spectrum of nutritional statuses, ranging from deficiency to adequate or even excessive intake, among OAs.

#### 3.2.3 Sex-related differences in energy and nutrient intake among OAs

As shown in Figure 2, in the TLGS cohort, the mean daily energy intake among men was 2076 kcal (95% CI: 2032.1, 2119.9), significantly higher than the intake reported for women (1818 kcal; 95% CI: 1781.2, 1854.8). This pattern was repeated in the BLAS cohort, where men repeated a mean daily energy intake of 2,555 kcal (95% CI: 2425.8, 2684.2), compared to women (2,387 kcal; 95% CI: 2282.1, 2491.9). Similar sex-based differences were observed for other macronutrients. The non-overlapping 95% confidence intervals for most comparisons between men and women within each cohort confirm that these differences are statistically significant. These findings indicate persistent sex-related disparities in dietary intake patterns among Iranian OAs across different geographical regions.

In the TLGS cohort, daily mean calcium intake was 959 mg for men and 892 mg for women. In the BLAS cohort, corresponding values were 1,079 mg for men and 979 mg for women. Zinc intake followed a similar pattern, with higher mean intakes among men in both TLGS (12.1 mg vs. 10.4 mg) and BLAS (13.2 mg vs. 12.3 mg).

Vitamin A intake in the TLGS cohort was relatively higher than that observed in other micronutrients, with mean values of 663 μg/day for men and 696 μg/day for women; however, despite these averages, a substantial proportion of participants in both sexes failed to meet recommended intake levels. Folate intake in the TLGS cohort was 504 μg for men and 437 μg for women. Sex-specific detailed nutrient intakes for TLGS and BLAS are available in Supplementary Table 2.

Clear sex-based differences in energy and macronutrient intake were observed in both cohort studies, with men consistently reporting higher intakes than women (Figure 2). Also, a persistent sex-related gap was observed across most nutrients in both cohorts. The observed gaps suggest that older women may be at greater risk of inadequate intake for several key nutrients.

## 4 Discussion

The present study provides a comprehensive, population-based description of the adequacy of food group and nutrient intake among Iranian OAs, using evidence from IHEIS and two cohort studies (TLGS and BLAS). To our knowledge, this is the first national-level attempt to estimate the adequacy of food group and nutrient intakes specifically among OAs in Iran. The findings revealed clear structural weaknesses in the dietary patterns of the elderly in Iran and indicated a level of nutritional inadequacy in this group that goes beyond minor deviations from the recommended intake.

### 4.1 Food groups intake

OAs exhibited a predominantly grain-based dietary pattern with limited diversity. This pattern is consistent with the macroeconomic context, as existing evidence indicates that lower Gross Domestic Product (GDP) is associated with reduced consumption of fruits and meats, while higher inflation is linked to an increased reliance on grains (such as bread, rice, and pasta) (16). A high reliance on such a calorie-dense but nutrient-poor dietary pattern can lead to deficiencies in high-quality protein, iron, and B vitamins, causing malnutrition and various diseases such as sarcopenia and other chronic diseases in OAs (17).

In both the IHEIS and BLAS datasets, intakes of fruits, vegetables, dairy products, and meat were below recommended levels, whereas only the TLGS cohort showed fruit and vegetable intakes exceeding reference values—likely reflecting structural differences in the demographic characteristics, food access, and socioeconomic profiles of participants in this cohort. Research across different regions has reported similar findings on food group intake among the elderly populations (18–20). The adherence to recommendations was associated with younger age, higher education, and health-promoting behaviors (21). The consistency of study results across regions, despite cultural and methodological differences, demonstrates that the elderly are at increased risk of aging-related nutrition-related problems (22). This process often involves declines in oral function, appetite, financial capacity, social engagement, and dietary autonomy, which together reduce dietary quality. Inadequate consumption of food groups places older individuals at risk of malnutrition, which is strongly associated with increased mortality and morbidity (23).

The World Health Organization (WHO) recommends a daily intake of 400 g of fruits and vegetables to reduce the risk of chronic diseases, including cardiovascular disease, cancer, diabetes, and obesity (24). Beyond reducing all-cause mortality, adequate fruit and vegetable intake among OAs is associated with lower rates of hypertension, atherosclerosis, stroke, and frailty (the latter through improvements in bone density and muscle strength). These effects are attributable to the high content of carotenoids, antioxidants, vitamins, and minerals of fruits and vegetables (25). Several factors have been associated with low fruit and vegetable consumption, including demographic characteristics, social factors, disease status, mental health, lifestyle behaviors, and cognitive and psychosocial factors (26–28). In Iran, higher age, greater education and welfare, lower prices, and greater accessibility have been directly linked to fruit and vegetable consumption among the elderly. Married OAs also consumed more vegetables than widowed or divorced individuals, confirming the role of family and social support in fruit and vegetable consumption among the elderly (29).

The United States Department of Agriculture (USDA) recommends consuming three cups of dairy products daily, including milk, yogurt, and cheese (30). In this study, dairy intake appears lower in older age groups, particularly among individuals aged ≥ 80 years. Preliminary evidence suggests an inverse association between low-fat milk consumption and the incidence of both frailty and Alzheimer’s disease (31). Low dairy product intake may be related to a variety of factors, including decreased personal health awareness, a higher prevalence of lactose intolerance and indigestion, changes in taste and palatability, attempts to reduce fat intake, age-related anorexia, or even financial constraints and the accessibility of dairy products (32).

Lean meat consumption is also emphasized in the dietary guidelines as part of a healthy dietary pattern (30). Meat is a dense source of essential nutrients—including protein, heme iron, vitamin B12, zinc, and specific fatty acids—that supports the maintenance of muscle mass and strength in aging populations (33). Evidence indicates that meat consumers show lower prevalence of micronutrient inadequacy (34). Therefore, adequate meat consumption could play a vital role in reducing malnutrition among the elderly. In Iran, economic conditions strongly influence the affordability and consumption of foods, including fish, meat, and dairy products as a dietary protein source, among the elderly (35).

Improving diet quality through food-based rather than nutrient-based approaches has demonstrated stronger and more consistent associations with reduced risk of adverse health outcomes (36). Diets that effectively prevent chronic diseases, such as diabetes, typically emphasize unprocessed foods from five core food groups: fruits, vegetables, grains, dairy, and meat or their alternatives. Consequently, chronic disease management guidelines in several countries recommend adherence to national food dietary guidelines structured around these food groups (37, 38).

### 4.2 Macro- and micronutrient intake

The macronutrient intake estimates derived from the two cohort studies differed substantially from those obtained through the IHEIS data. Mean energy intake was higher in the BLAS compared with the TLGS; however, the proportional contribution of protein to total energy intake was lower, and the share of fat was higher in the BLAS. The TLGS participants, in contrast, demonstrated a more favorable nutrient profile with higher fiber intake and lower saturated fat consumption. Notably, only the BLAS cohort reported energy intakes aligned with the estimated energy requirements of OAs. In contrast, the IHEIS data showed a declining trend in energy intake over the 4 years examined, a pattern observed for other macronutrients and fiber. The findings from the three sources reviewed in this study indicated heterogeneity in the nutritional intakes of the Iranian elderly. Heterogeneities in macronutrient intake observed across studies and regions may be attributable to broader socioeconomic shifts—including economic development, modernization, and urbanization—that influence food availability, dietary preferences, and consumption patterns across Iran (39, 40). Overall, the macronutrient profiles across the three data sources indicated a suboptimal dietary pattern among Iranian OAs (Table 1).

These findings are consistent with those of a study in Taiwan that examined trends in macronutrient intake among Taiwanese OAs (41). The study indicated a fluctuating trend in energy inadequacy among the elderly across survey cycles, alongside a decline in mean energy intake. Unlike the present study, in which the proportional contributions of carbohydrates, fats, and proteins remained fairly stable and largely within recommended ranges, the Taiwanese study showed increasing shares of these macronutrients over time. Identifying the causes of low energy intake in OAs is challenging due to inconsistent malnutrition data and frequent reliance on assumed body weights in analyses. These assumptions may have led to over- or underestimation of energy requirements in both sexes. Methodological considerations should also address energy underreporting in dietary assessments; underreporting among community-dwelling OAs has been estimated at 10–15% (42).

Despite meeting mean intake targets, the protein quality in this elderly population appears suboptimal due to low consumption of high-quality sources and a heavy reliance on cereals. This dietary pattern, coupled with evidence that current protein recommendations may be insufficient to counteract age-related muscle loss, raises concerns about the risk of sarcopenia (43). While total fat intake among Iranian OAs was within the recommended range, the fatty acid profile was imbalanced, indicating poor fat quality. Furthermore, although SFA intake was low, intake of unsaturated fats (MUFA/PUFA) was inadequate, likely due to high consumption of solid/hydrogenated fats and low consumption of fish, olive oil, and nuts (44). In addition, economic and availability constraints may further limit access to these foods.

This study also revealed that the mean dietary fiber intake was consistently below the adequate intake (AI) for all age and sex groups of OAs. This inadequacy likely reflects low consumption of whole grains, legumes, nuts, fresh fruits, and vegetables in the Iranian diet. Given that factors such as altered gastrointestinal motility, polypharmacy, and low-fiber dietary patterns contribute to constipation among OAs (45), increasing dietary fiber may offer symptomatic benefits. In the absence of strong age-specific evidence, adherence to the current AI for men and women is recommended (46).

The present findings demonstrated a heterogeneous pattern of micronutrient adequacy among Iranian OAs. Calcium intake in both cohorts exceeded the IHEIS; however, in neither study was the recommended level met. This aligns with the generally low consumption of dairy products across all three databases. A similar downward trend was observed for zinc, with insufficient intake documented across the datasets examined. Also, intakes of vitamins A, B_2_, and D were below the recommended levels in both the TLGS cohort and the IHEIS, although the values obtained for vitamins A and D in the TLGS were much higher than those in the IHEIS. Furthermore, vitamin B9 and B12 intakes in the IHEIS fell below recommended levels. This pattern is consistent with evidence from other countries as well. A study of OAs in Malaysian agricultural regions found that intakes of vitamins B2, B9, A, and D, as well as calcium, were significantly below national recommendations (47). This alignment was also observed in a study by Jafar et al. in both rural and urban areas (48). These findings demonstrated a significant risk of micronutrient deficiencies among OAs, which can lead to health issues.

The elevated calcium requirements after age 50—from 800 mg to 1,000 mg per day—reflect age-related declines in intestinal calcium absorption. Inadequate intake in older age is clinically relevant, as insufficient calcium intake is strongly associated with osteoporosis and its downstream consequences, which can be particularly debilitating in late life (49). Beyond calcium, zinc has emerged as a potential target micronutrient in mitigating age-related muscle loss and delaying the onset of physical functional decline and frailty. The concurrent inadequacy of calcium and zinc, as observed in this study, may accelerate the trajectory of frailty and impaired physical performance among OAs (50).

Inadequate intake of vitamins B9 and B12 can impair homocysteine metabolism, elevating a key risk factor for age-related vascular disease, cognitive decline, and osteoporosis (51–55). Numerous studies have documented an increase in folate and vitamin B12 deficiencies with advancing age (56). Atrophic gastritis—which affects more than 50% of OAs, with even higher prevalence in Asian populations—can impair vitamin B12 absorption (57). Inadequate folate intake among OAs may result from substantial losses during cooking, particularly boiling vegetables (with reductions of up to 80%), or from difficulty chewing grains, which serve as major dietary sources of folate (58). Dairy products and meat are the primary dietary sources of vitamin B12 (59); however, consistent with the present study’s findings, the dietary intake of these food groups among Iranian OAs appears insufficient. Public health interventions—ranging from targeted nutrition education to appropriate supplementation—are essential for mitigating the adverse health consequences of insufficient micronutrient intake.

### 4.3 Age-related energy and nutrient intakes

The dietary intake of Iranian OAs showed a clear age-related decline in both quantity and quality over the study period. Energy, macronutrients, and essential fatty acids decreased progressively, with the most severe reductions observed in the oldest age groups. Micronutrient intake was broadly inadequate, with severe and progressive deficiencies in vitamins A, D, B2, B9, and B12, as well as key minerals, among the elderly, particularly the oldest-old. Declining vitamin C levels further reflected low consumption of fruits and vegetables.

These findings align with results of a German study conducted among “young-old,” “old-old,” and “very-old” adults, in which the intake of several nutrients—including calcium among men, and fiber, calcium, and vitamins B1, C, and A overall—declined with increasing age (60). A large Chinese study of adults over 80 found similar patterns: widespread inadequacies in energy, protein, vitamins A, B2, and folate, with intakes declining with age—in line with the present findings (61). Overall, direct comparisons across studies are difficult due to methodological variations, including differences in dietary assessment tools and nutrient databases, as well as heterogeneity in study sample characteristics—such as age distribution, health status, nutritional risk, and activity levels—which may influence nutrient requirements and intake patterns.

### 4.4 Sex-specific patterns of nutrient intake and nutritional adequacy among Iranian OAs

The results of this study revealed a persistent pattern of nutrient inadequacy among Iranian OAs, as reflected in both IHEIS and TLGS/BLAS cohort data, which is frequently compounded by significant sex-based disparities. In IHEIS, a comparative analysis across years suggests that although men consistently consumed more energy and protein than women, the NAR for many micronutrients remains low in both sexes, with deficiencies generally more severe among women. Particularly notable are inadequate intakes of vitamins A, D, B2, B9, B12, and C, as well as key minerals such as calcium, phosphorus, and zinc, with intake levels of 30–50% of daily requirements. This pattern of insufficient intake persists consistently across all years examined.

The MAR index, an overall indicator of dietary adequacy, further underscores the poor nutritional status of Iranian OAs. Across all years, MAR values remained below 0.6 in men and 0.55 in women, indicating widespread inadequate nutrient intake and poor overall diet quality among Iranian OAs. The consistently downward trend in MAR over time reflects a gradual deterioration in dietary quality. A comparison of IHEIS findings with those from the TLGS and the BLAS offers a clearer picture of the gap between actual and optimal intake. In TLGS and BLAS, due to food-recall-based methods, energy, protein, and a large proportion of micronutrient intakes are reported to be significantly higher than in IHEIS. For instance, energy intake in the BLAS cohort exceeded 2,500 kcal for men and more than 2,300 kcal for women, whereas the IHEIS reported values below 1700 kcal for men and 1,500 kcal for women. Despite this discrepancy, even in these higher-quality studies, micronutrient deficiencies—particularly calcium, vitamin D, vitamins B9, and B12—are consistently observed.

Consistent with the findings of the present study, a cross-sectional survey conducted in Korea among older men and women found lower NAR levels for vitamin A, riboflavin, and calcium, and these inadequacies increased with age. In this population, the MAR index showed a marked decline in the oldest age group (62). Similarly, in a study of adults aged over 65 years from five regions of Uganda, NAR levels for vitamin A, vitamin B12, and calcium were notably low across all participants. The MAR index was about 60% (63).

The results of the present study showed that the consumption of nutrient-dense food groups was inadequate among Iranian OAs. Animal-source foods, such as meat and dairy, provide essential nutrients for optimal physiological function (64). Globally, low intake of vitamin and mineral-rich foods, including fruits and vegetables, is associated with a higher proportion of diet-related mortality (65). Several studies have emphasized that socioeconomic and demographic factors—such as age, place of residence, education level, and income—significantly affect the nutrient adequacy in the older population (66, 67). Consistent with these observations, the dietary pattern of Iranian OAs is primarily grain- and starch-based, likely due to region-specific socioeconomic and demographic factors.

The simultaneous use of three data sources—two well-established cohort studies, including TLGS in Tehran (the capital) and BLAS in eastern Iran, and the national IHEIS database—is one of this study’s most important strengths. This geographical and methodological diversity allowed for a multidimensional picture of the dietary intake patterns of Iranian OAs. Using individual-based data from the cohorts, alongside household-based IHEIS data, increased the analysis power and enabled comparisons between observed consumption patterns and estimates derived from dietary recall.

In contrast, the substantive differences among these three data sources—including data collection instruments, measurement level (individual versus household), and methods of estimating consumption—are key limitations, and direct comparisons of consumption values should be made with caution. The TLGS and BLAS are based on individual self-report instruments, whereas the IHEIS estimates household-level consumption from food purchases. The IHEIS data do not necessarily reflect actual household OA consumption and may underestimate or overestimate micronutrient intake. Also, the limited geographic coverage of the two cohorts limits the ability to fully generalize the results to the entire country. Other limitations of this study include common errors in dietary recall instruments, inconsistent data for some micronutrients, and a lack of direct information on contextual factors such as price, availability, and food preferences. The cross-sectional study design also makes it difficult to determine cause-and-effect relationships.

## 5 Conclusion

This study provides a comprehensive assessment of dietary intake patterns and nutrient adequacy in Iranian OAs, integrating national data (IHEIS) and two cohort studies (TLGS and BLAS). Findings indicated a significant gap between actual and recommended intakes, particularly for key micronutrients such as calcium, vitamin D, folate, vitamin B12, vitamin A, and zinc. Despite higher energy and macronutrient intakes in the cohort studies, overall diet quality remains poor. The predominant dietary pattern of Iranian OAs is based on grains and starches, and the consumption of nutrient-rich foods, including dairy, meat, fruits, and vegetables, is low. The decline in energy and nutrient intake with age and gender differences identifies vulnerable groups in the elderly population. The findings also highlight the role of socioeconomic, demographic, and regional factors in nutritional adequacy. Disparities among studies may highlight a concerning gap in dietary habits within the populations represented by IHEIS and BLAS, which may necessitate targeted public health interventions. Overall, the study results emphasized the need for targeted nutrition interventions, including nutrition education, improved access to nutrient-rich foods, and, where appropriate, supplementation programs, to reduce micronutrient deficiencies and support healthy aging. Implementing evidence-based policies tailored to the characteristics of the Iranian elderly population is essential for improving diet quality and reducing the risk of nutrition-related diseases.

## Data Availability

The datasets presented in this study can be found in online repositories. The names of the repository/repositories and accession number(s) can be found in the article/Supplementary material.
