# Supplementary material for: Dietary intake adequacy among Iranian older adults: evidence from national household survey data and two population-based cohorts
Source: Front Nutr. 2026 Jun 26;13:1808784. doi: 10.3389/fnut.2026.1808784 (PMC13349781; doi:10.3389/fnut.2026.1808784)
Supplement: Supplementary file 1 [file Table_1.DOCX]

| Supplementary Table 1. Mean daily intakes of energy, macronutrients, fiber, and micronutrients among Iranian older adults according to age group, based on data from the Iran Household Expenditure and Income Survey (IHEIS), 2019–2022. | | | |
| --- | --- | --- | --- |
| Nutrients | **60–74 y** | **75–84 y** | **≥85 y** |
|  | Mean (95% CI) | Mean (95% CI) | Mean (95% CI) |
| Energy (kcal) | 1467.3 (1460.1–1474.5) | 1425.2 (1410.4–1440.1) | 1301.4 (1278.1– 1324.7) |
| Carbohydrate (g) | 236.7 (235.6–237.9) | 232.1 (229.7– 234.5) | 213.7 (209.9–217.5) |
| Protein (g) | 50.0 (49.7–50.3) | 49.0 (48.5– 49.5) | 44.0 (43.2–44.8) |
| Fat (g) | 47.0 (46.7–47.3) | 45.0 (44.4– 45.6) | 40.0 (39.0–41.0) |
| Linoleic acid (g) | 12.1 (12.0–12.1) | 11.1 (11.0–11.3) | 9.8 (9.6–10.1) |
| Linolenic acid (g) | 0.5 (0.5–0.5) | 0.5 (0.5–0.5) | 0.4 (0.4–0.4) |
| Vitamin A (μg) | 277.0 (274.3–279.7) | 263.0 (258.5–267.5) | 231.7 (224.6–238.8) |
| Iron (mg) | 10.8 (10.7–10.9) | 10.4 (10.3–10.5) | 9.5 (9.3–9.7) |
| Calcium (mg) | 357.5 (355.4–359.6) | 349.0 (353.4–344.6) | 316.5 (309.6–323.4) |
| Phosphorus (mg) | 411.9 (409.2–414.6) | 400.0 (394.6– 405.4) | 358.0 (349.9–366.1) |
| Zinc (mg) | 3.6 (3.6–3.6) | 3.5 (3.4– 3.6) | 3.1 (3.0–3.2) |
| Vitamin B1 (mg) | 1.5 (1.5–1.5) | 1.5 (1.4– 1.5) | 1.4 (1.3–1.4) |
| Vitamin B2 (mg) | 0.9 (0.9–0.9) | 0.8 (0.8–0.8) | 0.8 (0.8–0.8) |
| Vitamin B3 (mg) | 18.4 (18.2–18.5) | 17.9 (17.7– 18.1) | 16.5 (16.2–16.8) |
| Vitamin B6 (mg) | 0.8 (0.8–0.8) | 0.8 (0.8–0.8) | 0.7 (0.7–0.7) |
| Vitamin B9 (μg) | 174.4 (173.2–175.6) | 167.1 (164.5– 169.7) | 152.4 (148.6–156.2) |
| Vitamin B12 (μg) | 1.1 (1.0–1.1) | 1.0 (1.0–1.0) | 1.0 (1.0–1.0) |
| Vitamin C (mg) | 31.9 (31.7–32.1) | 30.5 (30.2– 30.9) | 27.0 (26.4–27.6) |
| Vitamin D (μg) | 0.4 (0.4–0.4) | 0.4 (0.4–0.4) | 0.4 (0.3–0.4) |
| Fiber (g) | 9.3 (9.3–9.3) | 9.1 (9.0– 9.2) | 8.2 (8.0–8.4) |
| Values are presented as mean (95% confidence interval).  Participants were categorized into three age groups: 60–74 years (n=81,745), 75–84 years (n=21,728), and ≥85 years (n=7,292).  CI, confidence interval; IHEIS, Iran Household Expenditure and Income Survey | | | |

| Supplementary Table 2. Mean intakes of selected macro and micronutrients of Iranian men and women elderly based on IHEIS (Iran household expenditure and income) data, TLGS (Tehran lipid and glucose study) and BLAS (Birjand Longitudinal Aging Study).  This table shows the mean daily intake of energy and macro-and micronutrients in elderly Iranian male and Female based on IHEIS, TLGS, and BLAS data. | | | | | | |
| --- | --- | --- | --- | --- | --- | --- |
|  | **IHEIS** | | **TLGS** | | **BLAS** | |
| Nutrient | **Male** (n=53846) | **Female** (n=56919) | **Male** (n=857) | **Female** (n=982) | **Male** (n=636) | **Female** (n=689) |
|  | Mean (95% CI) | Mean (95% CI) | Mean (95% CI) | Mean (95% CI) | Mean (95% CI) | Mean (95% CI) |
| Energy (kcal) | 1505.7 (1496.9– 1514.5) | 1406.0 (1397.1– 1415.0) | 2076.0 (2032.1– 2119.9) | 1817.9 (1781.1– 1854.7) | 2555.3 (2425.8– 2684.8) | 2387.0 (2282.1– 2491.9) |
| Carbohydrate (g) | 243.5 (242.1– 244.9) | 227.4 (226.0– 228.8) | 326.0 (318.5– 333.5) | 277.5 (271.5– 283.5) | 372.0 (345.6– 398.4) | 342.8 (322.0– 363.6) |
| Protein (g) | 47.7 (47.4– 48.0) | 44.5 (44.2– 44.8) | 79.3 (76.6– 82.0) | 68.0 (65.8– 70.2) | 86.3 (82.1– 90.5) | 80.1 (76.7– 83.5) |
| Linoleic acid (g) | 12.3 (12.2– 12.4) | 11.3 (11.2– 11.4) | -- | -- | -- | -- |
| Linolenic acid (g) | 0.5 (0.5– 0.5) | 0.5 (0.5– 0.5) | -- | -- | -- | -- |
| Vitamin A (µg) | 172.9 (169.5– 176.3) | 162.4 (159.6– 165.2) | 663.4 (633.8– 693.0) | 695.5 (591.0– 800.0) | -- | -- |
| Iron (mg) | 10.7 (10.6– 10.8) | 10.0 (9.9– 10.1) | 20.3 (19.3– 21.3) | 18.7 (17.7– 19.7) | 21.1 (19.7– 22.5) | 20.0 (18.8– 21.2) |
| Calcium (mg) | 305.0 (302.4– 307.6) | 286.9 (284.2– 289.6) | 959.2 (932.0– 986.4) | 892.0 (867.0– 917.0) | 1079.2 (1023.9– 1134.5) | 979.4 (940.3– 1018.5) |
| Phosphorus (mg) | 345.5 (342.2– 348.8) | 324.9 (321.6– 328.2) | 1412.7 (1379.9– 1445.5) | 1228.6 (1201.4– 1255.8) | 1721.4 (1626.3– 1816.5) | 1581.2 (1504.5– 1657.9) |
| Zinc (mg) | 3.0 (3.0– 3.0) | 2.7 (2.7– 2.7) | 12.1 (11.5– 12.7) | 10.4 (10.0– 10.8) | 13.2 (12.5– 13.9) | 12.3 (11.7– 12.9) |
| Vitamin B1 (mg) | 1.5 (1.5– 1.5) | 1.4 (1.4– 1.4) | 2.0 (1.9– 2.1) | 1.5 (1.5– 1.5) | -- | -- |
| Vitamin B2 (mg) | 0.6 (0.6– 0.6) | 0.5 (0.5– 0.5) | 1.8 (1.7– 1.9) | 1.5 (1.5– 1.5) | -- | -- |
| Vitamin B3 (mg) | 18.8 (18.7– 18.9) | 17.6 (17.5– 17.7) | 21.3 (20.8– 21.8) | 18.2 (17.8– 18.6) | -- | -- |
| Vitamin B6 (mg) | 0.6 (0.6– 0.6) | 0.6 (0.6– 0.6) | 2.0 (1.9– 2.1) | 1.8 (1.7– 1.9) | 2.0 (1.9– 2.1) | 1.8 (1.7– 1.9) |
| Vitamin B9 (µg) | 180.2 (178.7– 181.7) | 166.4 (164.9– 167.9) | 503.8 (492.8– 514.8) | 436.8 (427.6– 446.0) | -- | -- |
| Vitamin B12 (µg) | 0.5 (0.5– 0.5) | 0.4 (0.4– 0.4) | 3.1 (2.9– 3.2) | 2.6 (2.5– 2.7) | 3.2 (3.0– 3.4) | 2.8 (2.7– 2.9) |
| Vitamin C (mg) | 32.2 (32.0– 32.4) | 30.6 (30.4– 30.8) | 136.1 (130.1– 142.1) | 141.8 (135.9– 147.7) | 107.5 (97.2– 117.8) | 90.7 (84.0– 97.4) |
| Vitamin D (µg) | 0.4 (0.4– 0.4) | 0.4 (0.4– 0.4) | 3.5 (2.9– 4.1) | 2.8 (2.4– 3.2) | -- | -- |
| Dietary Fiber (g) | 9.5 (9.4–9.6) | 8.9 (8.8–9.0) | 40.1 (38.8–41.4) | 34.5 (33.5–35.5) | 14.4 (13.3–15.5) | 13.4 (12.5–14.4) |
| Values are presented as mean (95% confidence interval). Estimates are shown separately for males and females. Nutrients unavailable in the original datasets are indicated by "--".  IHEIS, Iran Household Expenditure and Income Survey; TLGS, Tehran Lipid and Glucose Study; BLAS, Birjand Longitudinal Aging Study; CI, confidence interval | | | | | | |

| Supplementary Table 3. Nutrient Adequacy Ratio (NAR) and Mean Adequacy Ratio (MAR) by sex and year among Iranian older adults (2019–2022). | | | | | | | | |
| --- | --- | --- | --- | --- | --- | --- | --- | --- |
| Nutrient | 2019 | | 2020 | | 2021 | | 2022 | |
|  | **Male** (n=13292) | **Female** (n=14019) | **Male** (n=13086) | **Female** (n=13861) | **Male** (n=13479) | **Female** (n=14164) | **Male** (n=13990) | **Female** (n=14875) |
| Energy | 0.7 | 0.8 | 0.6 | 0.7 | 0.6 | 0.7 | 0.6 | 0.7 |
| Carbohydrate | 1.0 | 1.0 | 1.0 | 1.0 | 1.0 | 1.0 | 1.0 | 1.0 |
| Protein | 0.9 | 1.0 | 0.8 | 0.9 | 0.8 | 1.0 | 0.8 | 0.9 |
| Linoleic acid | 1.0 | 1.0 | 0.9 | 1.0 | 0.9 | 1.0 | 0.8 | 1.0 |
| Linolenic acid | 0.3 | 0.4 | 0.3 | 0.5 | 0.3 | 0.5 | 0.3 | 0.4 |
| Vitamin A | 0.2 | 0.2 | 0.2 | 0.2 | 0.2 | 0.2 | 0.2 | 0.2 |
| Iron | 1.6 | 1.0 | 1.0 | 1.0 | 1.0 | 1.0 | 1.0 | 1.0 |
| Calcium | 0.4 | 0.3 | 0.3 | 0.2 | 0.3 | 0.2 | 0.3 | 0.2 |
| Phosphorus | 0.6 | 0.6 | 0.5 | 0.4 | 0.5 | 0.5 | 0.4 | 0.4 |
| Zinc | 0.3 | 0.4 | 0.3 | 0.3 | 0.3 | 0.4 | 0.3 | 0.3 |
| Vitamin B1 | 1.5 | 1.0 | 1.0 | 1.0 | 1.0 | 1.0 | 1.0 | 1.0 |
| Vitamin B2 | 0.5 | 0.5 | 0.5 | 0.4 | 0.5 | 0.5 | 0.4 | 0.4 |
| Vitamin B3 | 1.3 | 1.0 | 1.0 | 1.0 | 1.0 | 1.0 | 1.0 | 1.0 |
| Vitamin B6 | 0.4 | 0.4 | 0.4 | 0.4 | 0.4 | 0.4 | 0.4 | 0.4 |
| Vitamin B9 | 0.6 | 0.6 | 0.4 | 0.4 | 0.4 | 0.4 | 0.4 | 0.3 |
| Vitamin B12 | 0.2 | 0.2 | 0.2 | 0.2 | 0.2 | 0.2 | 0.2 | 0.2 |
| Vitamin C | 0.4 | 0.4 | 0.4 | 0.4 | 0.4 | 0.4 | 0.4 | 0.4 |
| Vitamin D | 0.0 | 0.0 | 0.0 | 0.0 | 0.0 | 0.0 | 0.0 | 0.0 |
| MAR | 0.6 | 0.6 | 0.6 | 0.6 | 0.6 | 0.5 | 0.6 | 0.5 |
| NAR, Nutrient Adequacy Ratio; MAR, Mean Adequacy Ratio.  NAR was calculated as the ratio of nutrient intake to the corresponding recommended intake. Values greater than 1.0 indicate intakes exceeding the recommended level. Estimates are presented separately for males and females for each survey year.  MAR was calculated as the mean of NAR values across all assessed nutrients. | | | | | | | | |
